# Poliovirus intrahost evolution is required to overcome tissue-specific innate immune responses

**DOI:** 10.1038/s41467-017-00354-5

**Published:** 2017-08-29

**Authors:** Yinghong Xiao, Patrick Timothy Dolan, Elizabeth Faul Goldstein, Min Li, Mikhail Farkov, Leonid Brodsky, Raul Andino

**Affiliations:** 1 Department of Microbiology and Immunology, University of California, San Francisco, CA 94158 USA; 2 Department of Biology, Stanford University, Stanford, CA 94158 USA; 3 Tauber Bioinformatics Research Center and Department of Evolutionary & Environmental Biology, University of Haifa, Mount Carmel, Haifa 31905 Israel

## Abstract

RNA viruses, such as poliovirus, have a great evolutionary capacity, allowing them to quickly adapt and overcome challenges encountered during infection. Here we show that poliovirus infection in immune-competent mice requires adaptation to tissue-specific innate immune microenvironments. The ability of the virus to establish robust infection and virulence correlates with its evolutionary capacity. We further identify a region in the multi-functional poliovirus protein 2B as a hotspot for the accumulation of minor alleles that facilitate a more effective suppression of the interferon response. We propose that population genetic dynamics enables poliovirus spread between tissues through optimization of the genetic composition of low frequency variants, which together cooperate to circumvent tissue-specific challenges. Thus, intrahost virus evolution determines pathogenesis, allowing a dynamic regulation of viral functions required to overcome barriers to infection.

## Abstract (web summary)

RNA viruses, such as polioviruses, have a great evolutionary capacity and can adapt quickly during infection. Here, the authors show that poliovirus infection in mice requires adaptation to innate immune microenvironments encountered in different tissues.

## Introduction

RNA viruses exist as dynamic and diverse populations that rapidly adapt to selective pressures. Viral genetic structure is shaped by constant mutation and selection^1, 2^. Diversity, which powers evolution, accumulates by misincorporation of nucleotides during RNA synthesis. However, diversity comes at a cost, since most mutations are deleterious. As lineages carrying beneficial mutations arise in the population, they accumulate detrimental mutations which prevent them from being effectively selected. This increasing mutational load slows the rate of evolution, an effect known as “Muller’s Ratchet”. Competition between adaptive lineages, or “clonal interference’, also slows the rate of evolution^3–8^. Without a mechanism to break the linkage between detrimental and beneficial mutations^9–11^ and avoid competition between beneficial mutations^3–8^, the rate of population evolution is greatly reduced^3–11^. Recombination accelerates the rate of evolution by mitigating the mutational load carried by adaptive lineages, relieving competition by purging deleterious mutations, and combining beneficial mutations into the same genome^4, 6^. Effective recombination is critical to rapid adaptation to dynamic selective environments.

The immune system represents a major barrier to viral infection. Innate immune responses exert strong selective pressures as the virus spreads throughout the organism. Infection sets forth a cascade of intracellular and extracellular innate immune signals that establish an antiviral state in the host^12–20^. Virus replication and intrahost spread depends on a virus’ ability to overcome early innate immune responses^14–16^. The type I interferon (IFN) response, for example, has been shown to control poliovirus tissue tropism in mice^12, 13^. As evidence of the strong selective pressure that innate immunity places on viral populations, viruses have evolved many mechanisms to directly counteract these signals and responses^17–20^.

Although the fitness of a virus, its ability to infect, propagate and spread to a new host, has been linked to the genetic diversity of the virus population^1, 2, 21–24^, the mechanistic links between diversity, adaptation, pathogenesis and in vivo selective pressures are not well understood. Given that innate immune responses represent the first line of defense against viral infection and certain immune effectors, such as IFN, elicit tissue-specific responses^12, 13^, we hypothesized that the virus’ ability to establish infection may be tied to its capacity to rapidly adapt to tissue-specific innate immune environments. In this study, we use poliovirus as a model to examine these questions and to establish whether and how a virus’ ability to adapt facilitates viral spread and pathogenicity.

## Results

### G64S/D79H(GD) confers lower mutation and recombination rates

**Fig. 1:**
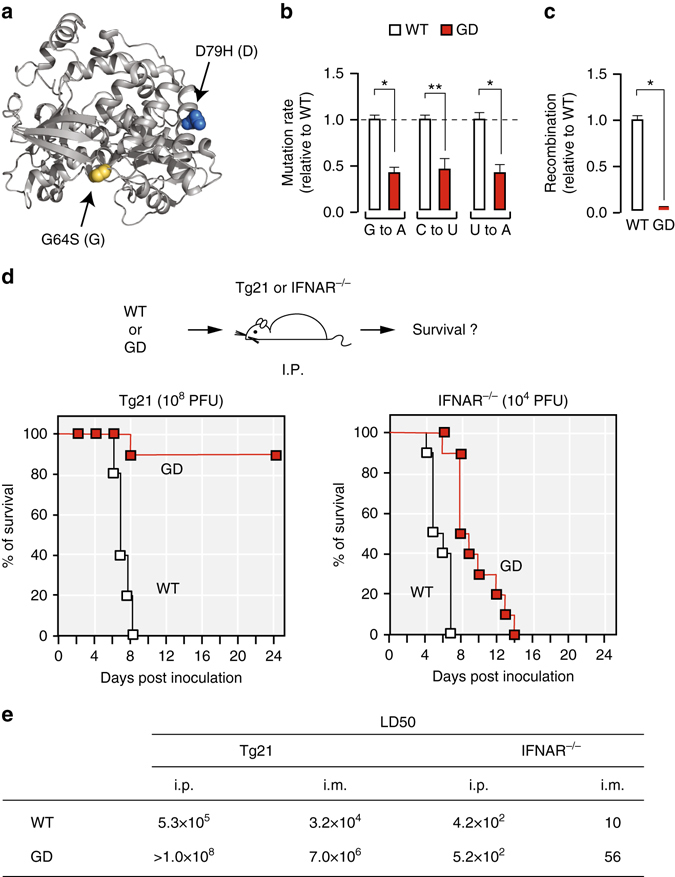
RNA viral evolution capacity is required to overcome the type I interferon (IFN) response. **a** The structure of RNA dependent RNA polymerase (RdRp) of poliovirus. G64S (G) confers higher replication fidelity and D79H (D) reduces recombination rate^24–26^. **b** Mutation rates associated of wild type (WT) and G64S/D79H (GD) poliovirus. Mutation rates are shown for G to A, C to U and U to A substitutions, based upon the frequency of lethal mutations (*P* < 0.05, seven passages, *n* = 7, mean ± s.d.). **c** Recombination rates for WT and GD poliovirus. For additional details see Supplementary Fig. 1. **d** Percentage survival of Tg21 mice or IFNAR^−/−^ mice (*n* = 10 for each cohort) infected with virus by I.P. inoculation route. Mice were inoculated with 10^8^ plaque-forming units (PFU) per Tg21 mouse or with 10^4^ PFU per IFNAR^−/−^ mouse (*n* = 10 for each cohort). **e** 50% lethal dose (LD50) by intra-peritoneal (I.P.) and intra-muscular (I.M.) route (*n* = 10 for each cohort)

To examine the role of mutation and recombination in vivo, we employed a poliovirus (PV) variant, G64S/D79H (GD), that carries two mutations within the RNA-dependent RNA polymerase (RdRp), 3D, which alter mutation (G64S)^24, 25^ and recombination (D79H) rates (Fig. 1a–c)^26^. Using a highly accurate deep-sequencing approach, CirSeq^27, 28^, which enables detection of very low frequency variants, we examined mutation rates of GD in HeLa cells. Our measurements confirmed that GD displays a 3-fold lower mutation rate compared with wild type (WT) (Fig. 1b, *P* < 0.05, *n* = 7). Using a recently developed cell-based recombination assay^26, 29^, we confirmed that recombination rate was reduced 10-fold in GD compared to WT (Fig. 1c and Supplementary Fig. 1).

### Adaptive capacity increases PV’s ability to overcome the Type I IFN response

With a higher mutation rate and efficient recombination, WT populations can take maximal advantage of rare beneficial mutations without being overwhelmed by an increasing load of deleterious mutations (Muller’s ratchet) or impeded by clonal interference^3–8^. GD carries defects in these two important evolutionary determinants. As such, we expect GD to be substantially limited in its capacity to rapidly adapt to selective pressures. To examine the hypothesis that rapid viral adaptation is required to overcome in vivo selective pressures, such as innate immune responses, we compared the outcome of infection with WT and GD viruses in a murine model. Immune-competent mice (Tg21) or mice defective in type I IFN receptor (IFNAR^−/−^), were infected with GD or WT populations by intraperitoneal (I.P.) or intramuscular (I.M.) routes (Fig. 1d and Supplementary Fig. 2). GD was significantly attenuated compared to WT in Tg21 mice (Log-rank (Mantel–Cox) test, *P* < 0.0001, *n* = 10, Fig. 1d and Supplementary Fig. 2, left panel). Irrespective of the route of inoculation, the 50% lethal dose (LD50) of GD virus is approximately 200-fold higher than WT virus in Tg21 mice (*n* = 10) (Fig. 1e and Supplementary Fig. 2). In the immune-compromised IFNAR^−/−^ mice, GD was pathogenic; suggesting that a major determinant for the observed GD attenuated phenotype in immune-competent mice is its inability to overcome type I IFN responses (Fig. 1d, *n* = 10, and Supplementary Fig. 2, right panel). Notably, even in IFNAR^−/−^ mice, the virulence of GD is slightly reduced, as mouse survival is extended by a few days compared to WT. Thus, although type I IFN response is one major factor determining GD attenuation, additional selective pressures are likely to determine the dynamics of infection in immune-competent mice.

To test virus replication kinetics, we compared the growth of GD virus to WT in cultured cells. GD virus production and RNA replication rate was not significantly different from that of WT in L20B, a murine fibroblast cell line (Supplementary Fig. 3 and Methods). Additionally, we performed a more sensitive competition assay to calculate the viral fitness (Supplementary Fig. 3), where L20B cells were infected with GD and WT at a 1 to 1 ratio. Following four passages, GD and WT maintained the 1 to 1 ratio (Supplementary Fig. 3C). Thus, while GD replication fitness is similar to WT in murine cells in culture, the low diversity GD virus is unable to effectively replicate and spread in immune-competent mice. Together these observations are consistent with the idea that rapid adaptation is required to overcome the diverse challenges encountered in vivo, such as innate immune responses.

**Fig. 2:**
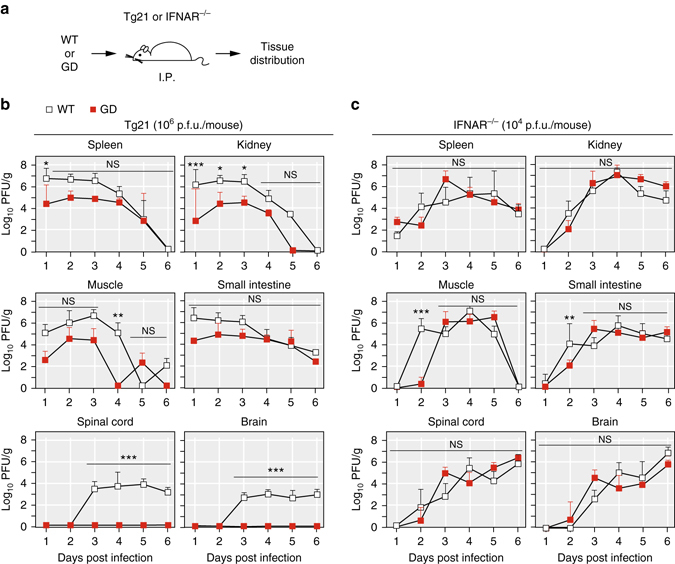
The tissue distribution of the virus strains in Tg21 and IFNAR^−/−^ mice. **a** Experimental schematic for determining the virus titer in individual tissues. **b**, **c** Virus titers in tissue collected from WT or GD infected mice by I.P. inoculation route. Tg21 (10^6^ PFU per mouse) or IFNAR^−/−^ (10^4^ PFU per mouse). Data are shown as logarithm, mean ± s.d., PFU per gram of tissue (*n* = 5, the number of mice is five for each time point and each group). Student’s *t*-test, n.s. indicates *P* > 0.05, *P ≤ 0.05, **P ≤ 0.01, ***P ≤ 0.001. Limited detection level is 20 PFU per gram tissue

To further test this idea, we examined the tissue-specific replication levels of the viruses in Tg21 and IFNAR^−/−^ mice infected with GD or WT virus (Fig. 2). WT virus inoculated to immune-competent mice was readily detected in spleen, kidney, small intestine, muscle, spinal cord and brain of immune-competent mice (Fig. 2b). GD virus was also detected in spleen, kidney, small intestine, muscle, albeit to lower levels, but remained undetectable in spinal cord and brain in immune-competent mice, even at 6 days postinfection (Fig. 2b). In contrast, in IFNAR^−/−^ mice, WT and GD virus populations replicated with nearly identical kinetics in spleen and kidney, reaching similar titers and quickly spreading to the central nervous system (CNS) (Fig. 2c). If delivered intra-cranially, GD can replicate in the CNS of immune-competent Tg21 mice (Supplementary Fig. 4), suggesting that the attenuated phenotype of GD in Tg21 mice is not due to the inability of GD to replicate in CNS, but rather to overcome the IFN-induced barriers to establish infection in the CNS. We thus conclude that WT virus, with full adaptive capacity, is able to overcome these innate immune challenges more effectively, while GD, with a limited capacity to adapt, cannot and is thus restricted to replicate at lower levels in fewer tissues.

### Tissue-specific responses induced during PV infection

**Fig. 3:**
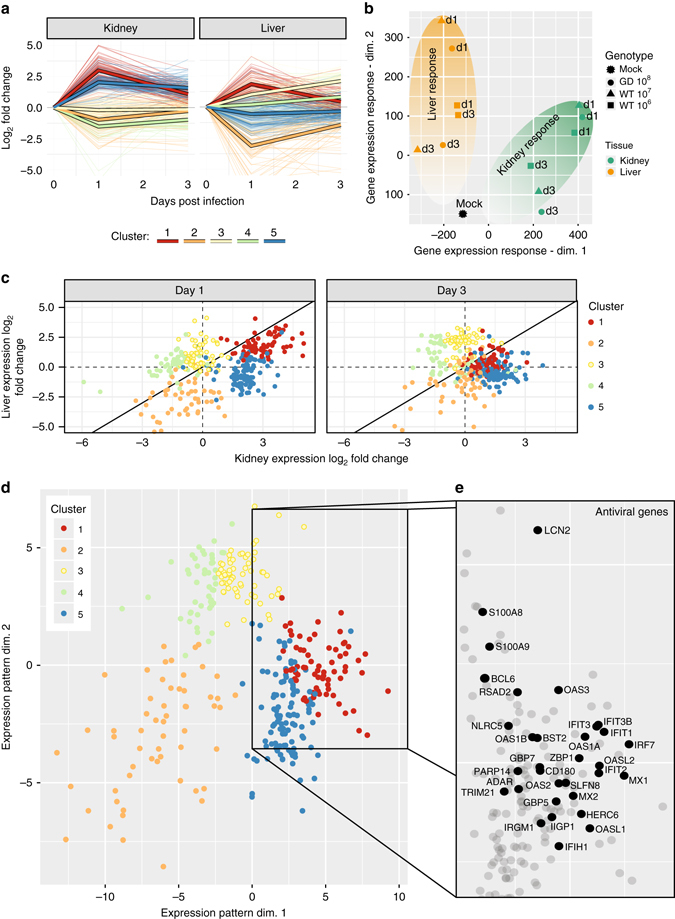
Tissue-specific transcriptional responses induced by poliovirus infection. **a** Line plot showing the gene expression profiles for 341 genes in liver and kidney from Tg21 mice infected with WT poliovirus at 10^7^ PFU by I.P. inoculation route. (*n* = 3, the number of mice is three for each time point and each group). Gene expression profiles across all experimental conditions were clustered to yield 5 clusters (Methods). Each gene’s trajectory is shaded by its cluster and the mean trajectory for each cluster is shown as a thick line. **b** Multidimensional scaling plot of the transcriptional response trajectories in liver and kidney. Each point represents the mean transcriptional response of three mice. Shaded ovals are drawn to emphasize the distinct trajectories of liver and kidney responses in infected mice. **c** Scatter plot showing the relative induction of genes in liver and kidney at 1 (left) and 3 (right) days postinfection. Genes are colored by cluster. **d** A multidimensional scaling plot of the coregulatory relationships between genes. Genes are arranged by the similarity of their expression response (determined by Euclidian distance) and colored by cluster. **e** Known innate immune and antiviral genes group together in clusters 1 and 5

Cellular environments and antiviral responses vary between tissues^12^. We hypothesized that these variable environments exert distinct evolutionary constraints on infecting viral populations and, thus, the virus population needs to adapt to the specific environment to replicate efficiently. To quantify the response to viral infection in individual tissue, we performed genome-wide transcriptome analysis using RNA-Seq (Methods). We focused on liver and kidney because both WT and GD viruses replicate in these tissues, allowing to link adaptation capacity and tissue specific responses to infection. Livers and kidneys were collected from immune-competent Tg21 mice (*n* = 3 for each time point) after infection with 10^6^ or 10^7^ PFU of WT virus, or 10^8^ PFU of GD virus, administered by I.P. route at 1 and 3 days postinfection. Genes which were altered in expression by greater than 4-fold in response to infection (with a false discovery rate, *q* < 0.01) at day 1 or day 3 postinfection in either tissue were used for clustering (*N* = 341) to identify the genes with similar transcriptional profiles. As expected, the infected mice show marked alterations in gene expression in both liver and kidney when compared to mock-infected mice (Fig. 3). Genes in two clusters show increased expression level at day one postinfection, clusters 1 and 5 (Fig. 3a, c). Cluster 5 shows a largely kidney-specific induction in response to infection. Conversely, the expression of the genes in Cluster 2 were downregulated in response to infection in liver (Fig. 3a, c). To visualize the global differences in the dynamic transcriptional response to infection in kidney and liver during infection, we used multidimensional scaling to compare global gene expression in each sequenced tissue (Fig. 3b). This visualization emphasizes the tissue-specific responses to poliovirus infection in liver and kidney and highlights the dynamics of the host responses. Gene expression responses at day 1 show the greatest difference compared to mock and these responses begin to recover to control gene expression levels by day 3.

**Fig. 4:**
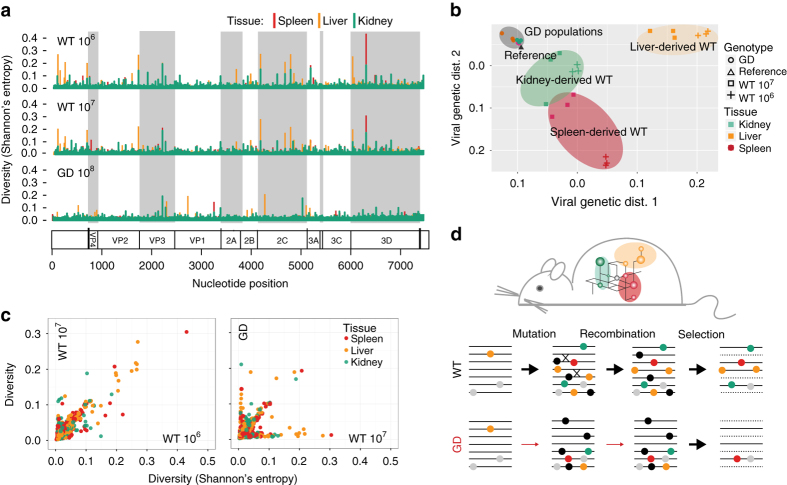
Genetic composition of poliovirus promotes intrahost evolution through counteracting tissue specific interferon responses. **a** Tissue-specific patterns of diversity in organ-resident populations. Virus was isolated at 3 days postinfection in Tg21 mice (*n* = 3, the number of mice is three for each time point and each group). Liver (LV), kidney (KD), and spleen (SP). Diversity is represented as Shannon’s Entropy. **b** The genetic structures of in vivo adapted populations. Multidimensional scaling correlation. In vivo adaptation leads to tissue-specific genetic structures (*colored ovals*) in WT populations from LV, KD and SP, but not in GD populations (*grey oval*). **c** Correlation plot of diversity between tissue-specific adapted populations between viral strains. **d** Model highlighting how efficient recombination and mutation leads to rapid adaptation during intrahost spread. G64S/D79H(GD), engineered to be deficient in recombination and diversity, is limited in its ability to rapidly adapt to changing selection pressures

Importantly, the transcriptional responses to infection were very similar between virus genotypes, indicating that, at the titers used in these experiments, GD and WT encounter similar levels of tissue specific factors and, as a result, similar levels of selection as defined by these factors (Fig. 3c and Supplementary Fig. 5). This is consistent with the observation that similar levels of virus were detected at 1 day postinfection with 10^7^ PFU of WT virus and 10^8^ PFU GD per mouse in the spleen and kidney of the infected mice, suggesting that, despite different initial titers, these viral genotypes elicit very comparable responses to infection (Supplementary Fig. 5 and 6).

We next examined the pattern of the expression for known immune genes and bona fide anti-poliovirus genes^30^. These genes show striking similarity in their transcriptional responses, grouping together in clusters 1 and 5 (Fig. 3d,e), emphasizing their tight co-regulation during infection. Most of these genes, including *IRF7*, *MX1*, *MX2*, and a number of *OAS* paralogs fell into the highly upregulated cluster 1, which shows greater upregulation in kidney, while others, including *ADAR, TRIM*, and *CCL9*, fall in cluster 5, which shows higher kidney-specific expression (Fig. 3c). It is likely that these differences in the levels of the induction of antiviral genes result in distinct selective environments in the kidney and liver.

### The virus population structure is determined by the tissue-specific environment

Given that global transcriptional responses to infection are distinct and specific to a given tissue type (Fig. 3), we next examined the hypothesis that these unique antiviral microenvironments drive tissue-specific intrahost adaptation. Tg21 mice were infected with WT and GD and viral populations were isolated from spleen, kidney and liver and analyzed by deep sequencing to assess the tissue-specific diversity and genetic structure. WT populations isolated from the same infected tissues showed strikingly similar patterns of mutation composition across the genome (Fig. 4a–c). In contrast, GD populations isolated from infected tissues showed similar overall diversity, however, the pattern of diversity differed from WT populations. (Fig. 4a, b). For example, liver-derived WT populations accumulated mutations at sites in the 5′ UTR and in the 2C coding region of the PV genome and the majority of mutations in spleen-derived populations accumulated in the 3Dpol protein. In contrast, these mutations were not observed in the GD populations. All three populations exhibited similar patterns in the VP3 coding region (Fig. 4a).

**Fig. 5:**
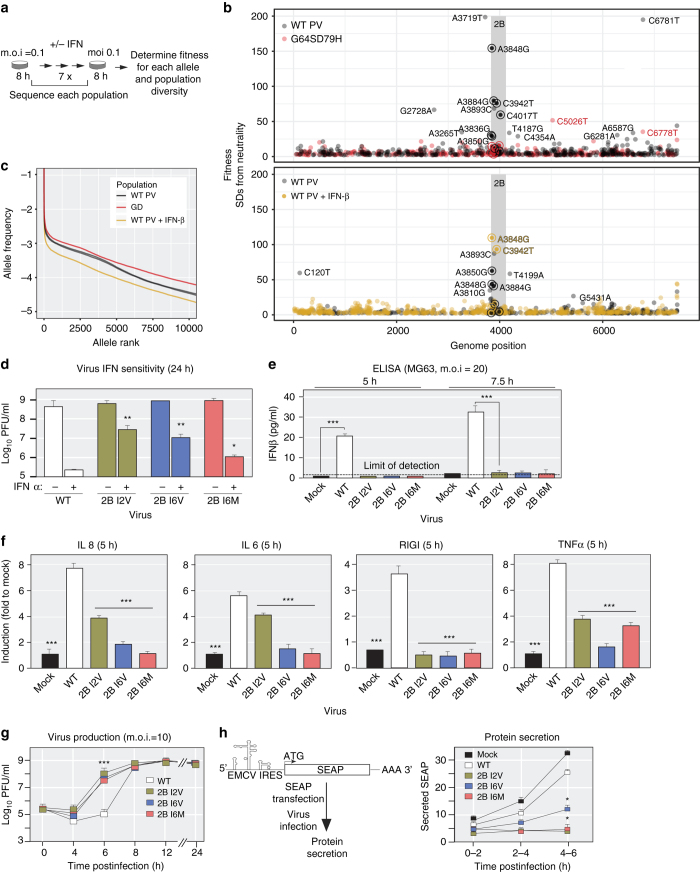
The 2B coding region of the poliovirus genome is a hotspot for the accumulation of mutations following cell culture adaptation. **a** High-density genetic screen procedure to identify adaptive mutations on cell culture conditions. WT, GD, or WT were passaged in HeLa cells or HeLa cells pre-treated with interferon β (IFN β). Using circular resequencing (CirSeq), the mutation composition, allele frequency distribution, and fitness of specific alleles were determined. **b** Scatter plot showing the position and fitness of identified adaptive mutations. Adaptive alleles with fitness values >2 standard deviations (s.d.s) above neutrality are shown. Alleles with fitness > 25 s.d.s above neutrality are labeled. The details of the specific mutations labeled in the plot are shown in Supplementary Table 1. The 2B coding region of the genome is shaded grey. **c** Allele frequency spectrums for 4 independently passaged of poliovirus (PV) populations. Alleles are ranked by frequency and plotted to show the relative diversity of passaged populations. All populations shown are from passage 6 of parallel passage experiments. **d** Interferon (IFN) sensitivity assay for specific 2B mutations identified in the passage screen. One-tailed Student’s *t*-test. *P ≤ 0.05, **P ≤ 0.01, *n* = 3. Data were presented as logarithm(mean ± s.d.), PFU per ml. *n* = 3, three replicates for each time, for each viral strain. **e,f** Comparison of immune signaling in MG63 cells infected by WT or 2B mutants at m.o.i=20. **e** Supernatant was collected at the indicated times to measure IFN β induction by ELISA. IFN β level < 2.3 pg /ml is undetectable. **f** mRNA expression level of *IL6, IL8, RIG-I, TNFα* measured by qRT-PCR at postinfected 5 h by 2B mutants and WT. Data shows mean ± s.d., * *P* < 0.05, ** *P* < 0.01, *n* = 3, Student’s *t*-test. **g** Growth kinetics of poliovirus carrying 2B mutations identified in HeLa cells. Viruses replication was examined for 2B mutants and WT by one-step growth curves in HeLa cells (m.o.i=10). (Data are shown as mean ± s.d., ***P < 0.0001, *n* = 3, three replicates for each time, for each viral strain, Student’s *t*-test). **h**. SEAP reporter assay to assess the ability of 2B mutants to inhibit protein secretion activity. One-tailed Student’s *t*-test. *P ≤ 0.05, *n* = 3, three replicates for each time, for each viral strain

We next used multidimensional scaling to visualize the pairwise genetic distances between the sequenced viral populations isolated from each tissue. Spleen-, liver- and kidney-derived WT populations formed distinct genotypic clusters, indicating that WT populations rapidly establish tissue-specific population structures in vivo (Fig. 4a, b). Importantly, the WT populations isolated from different mice clustered together by tissue, indicating that these population structures are established reproducibly during infection in independent, isogenic hosts (Fig. 4c). In contrast, GD virus isolated from all tissues remained nearly identical to the parental genotype, consistent with the idea that GD reduced evolutionary capacity precludes the virus to evolve in a given environment (Fig. 4a–c).

GD is highly attenuated in the presence of an intact immune response, but this attenuation is largely relaxed in IFNAR^−/−^ mice. Our RNA-Seq data indicate that the response to infection, including innate antiviral responses, vary between tissues. Population sequencing reveals that WT populations establish specific population structures in vivo, presumably adapting to tissue-specific selection. On the contrary, while GD elicits a similar antiviral response in infected tissues it cannot establish a successful infection in certain tissues and is cleared from tissue more rapidly than WT (Fig. 2). Taken together, these data suggest a model for the attenuation observed in GD infected mice (Fig. 4d). In WT infection, mutation and recombination together facilitate the establishment of tissue-specific population diversity. However, GD, with defects in mutation and recombination, is less effective in establishing tissue-specific population compositions and therefore cannot overcome the microenvironment of antiviral selection (Fig. 4d). This raises the question of how specific patterns in the population composition, consisting of low frequency alleles, can counteract antiviral defenses, and contribute to overcoming barriers to infection.

### The 2B coding region is a hotspot for the accumulation of adaptive mutations

In vivo selective environments are highly complex. A given tissue is constituted by multiple cell types. Physicochemical environments, macromolecular compositions, and antiviral responses all vary between tissues. In addition, the dynamics of populations during intrahost spread, characterized by small population sizes and repeated bottlenecking are not well defined, cannot be controlled, and may result in stochastic processes such as genetic drift, affecting the mutation composition of the population. Thus, to better understand the mechanisms and the population dynamics contributing to viral fitness and adaptation, we turned to a simple and well controlled, cell culture system, which circumvents many of the confounding factors associated with infection in animals.

We carried out seven serial passages in HeLa cells, using WT and GD viral populations at a multiplicity of infection (m.o.i) of 0.1, limiting each passage to a single replication cycle 8 h (schematized in Fig. 5a and Methods). The mutation composition of each population was analyzed by an ultra-accurate next-generation sequencing technique, CirSeq, which allowed us to identify very low-frequency mutations^27, 28^.

Similar to the tissue-specific patterns of diversity observed in vivo, WT populations accumulated reproducible cohorts of high fitness mutations. Adaptive mutations were distributed along the viral genome, strikingly, a cluster of mutations accumulated within the 2B protein coding region in multiple WT passage experiments (Fig. 5b and Supplementary Table 1). Protein 2B is a small, multifunctional membrane protein consisting of only 97 amino acids, which may function both as an independent protein and as an un-cleaved 2BC precursor^31^. In contrast, GD passaged in parallel demonstrated a reduced accumulation of adaptive mutations at any particular region in the genome (Fig. 5b and Supplementary Table 1), which is similar to our observations in vivo.

These observations are consistent with the idea that GD is defective in its ability to adapt, and thus cannot accumulate adaptive mutations to overcome tissue-specific challenges (Fig. 4b). Examining the allele frequency spectrum of the passaged populations provides an alternative view of the effects of selection, mutation and recombination on the populations (Fig. 5c). WT populations (Fig. 5c, WT and PV in black) show remarkably similar distributions of allele frequency, while the number of low frequency alleles is higher in the passaged GD population, suggesting that GD population is defective in accumulation and selection of mutations. This increase in low frequency mutations but apparent lack of adaptive alleles (Fig. 2b) is likely due to the “Muller’s ratchet” effect on the population.

As type I IFN responses appear to be a major obstacle for GD in establishing a robust infection in vivo, we next examined the virus adaptation to an IFN-activated environment. WT virus was serially passaged in HeLa cells pre-treated with IFNβ or untreated control Hela cells (Fig. 5a). Strikingly, several 2B mutations sharply increase their fitness value under IFN pre-treatment condition (Fig. 5b). Fewer adaptive alleles were identified under IFN treatment, and the population diversity of these populations was more limited (Fig. 5c), consistent with stronger purifying selection due to IFN-induced immune responses.

The information obtained from infected animals (Figs. 1–4) suggests that virus adaptation is required to overcome innate immune barriers and thus establish a successful infection (Figs. 3 and 4). Our in vitro experiments identified a hotspot in the 2B protein region for adaptive alleles that appear to be selected when cells are stimulated with IFNβ. Importantly, these mutations are not selected in GD, consistent with the idea that GD exhibits a reduced capacity to evolve in response to selection. To directly test whether the mutations observed in 2B counteract innate immune signaling, we engineered six of the high fitness 2B mutations into the PV genome. HeLa cells, pretreated with 500 IU/ml IFN alpha (IFNα) for 24 h, were infected with 2B variants or WT virus. Three of 2B mutants: 2B I2V (amino acid 1032 Ile->Val, nucleotide position, 3836–3838 ATC to GTG), 2B I6V (amino acid 1036 Ile->Val, nucleotide position, 3848 A->G) and 2B I6M (amino acid 1036 Ile->Met, nucleotide position, 3850 A->G) yield at least 100 times more virus than WT in IFNα pretreated condition (Fig. 5d) (Student’s *t*-test, *p* < 0.05, *n* = 3). These results suggest that alleles identified in 2B overcome restrictions on replication imposed by the effects of IFN response.

### Inhibition of the antiviral IFN system by 2B variants

To further study the effect of 2B variants on the IFN signaling, we initially compared IFN β production following WT or the 2B mutants infection. MG63 cells^32^ infected with WT produced readily detectable levels of IFNβ at 5 and 7.5 h postinfection (Fig. 5e). In contrast, infection with 2B mutants did not induce detectable levels (<2.3 pg per ml) of IFNβ (Fig. 5e). Furthermore, genes, *RIG-I*, *TNFα*, Interleukin 6 (*IL6*), and Interleukin 8 (*IL8*) were all expressed at significantly lower levels in cells infected with 2B mutants compared to WT virus (Fig. 5f) (Student’s *t*-test, *p* < 0.001, *n* = 3).

Together, these experiments indicate that mutations accumulating within 2B counteract innate immune responses in cell culture system. The viral protein 2B has been implicated in poliovirus RNA synthesis, inhibits protein secretion and membrane permeability^33, 34^. It is possible that the increase in IFN suppression is the consequence of an increase of virus replication or a direct effect on IFN secretion. To examine if the 2B mutants enhance viral replication, we examine virus production by performing one-step growth curve. A significant increase in viral replication rate was observed for 2B I2V, 2B I6V, and 2B I6M compared to WT at 6 h postinfection. However, by 8 h, 2B variants and WT are produced at similar levels (Fig. 5g).

Next we examined the effect of 2B mutations on protein secretion. We transfected an EMCV-IRES-driven secreted alkaline phosphatase (SEAP) reporter into COS7 cells^33, 35^ (Fig. 5h), then infected COS7 cells with 2B mutants or WT virus. We measured SEAP activity accumulating in the media of infected cells between 0 and 2, 2 and 4, and 4 and 6 h postinfection. The 2B mutants inhibited protein secretion to a greater extent than WT (Fig. 5h) (One-tailed Student’s *t*-test, *n* = 3, *p* < 0.05), indicating that the mutations in 2B may modulate its effect on cellular protein secretion.

### Effect of 2B mutants on viral replication and pathogenesis in mice

**Fig. 6:**
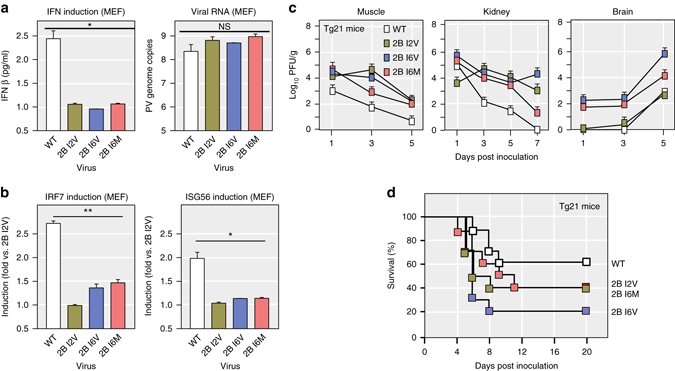
Adaptive 2B mutants increased virus replication and pathogenesis immune-competent mice. **a**, **b** The adaptive 2B mutants induce less IFN response in primary murine embryo fibroblast cells (MEFs). **a** MEFs were infected with WT and 2B mutants at m.o.i=0.1. supernatant was collected to measure IFNβ by ELISA at 72 h postinfection. RNA copy number of poliovirus genome was measured by qRT-PCR on MEFs at postinfection 72 h. **b** mRNA expression level of *IRF7* and *ISG56* was measured by qRT-PCR on MEFs at postinfection 72 h (*n* = 3, three replicates for each time, for each viral strain. Student’s *t*-test). n.s. indicates *P* > 0.05; *P ≤ 0.05, **P ≤ 0.01. **c** Virus tissue distribution. Viral titers in kidney, muscle and brain of Tg21 mice inoculated with 10^6^ PFU of WT, 2B I2V, 2B I6V, and 2B I6M administered I.P. route. Data were presented as logarithm(mean ± s.d.), PFU per ml. *n* = 4, the number of the mice is four per time point per viral strain. Limited detection level is 20 PFU per gram tissue. **d** Survival curve of Tg21 mice Tg21 mice were injected with 10^7^ PFU WT, 2B I2V, 2B I6V, or 2B I6M administered by I.P. route. (*n* = 10, the number of the mice is ten per group)

We next tested if the adaptive 2B mutants, identified in HeLa cells, also counteract the type I IFN response in primary murine embryo fibroblast cells (MEFs) isolated from immune-competent Tg21 mice. MEFs were infected with 2B mutants or WT virus and the level of secreted IFNβ was measured at 72 h postinfection. Although the same levels of viral RNA were detected at 72 h postinfection, the 2B mutants induced significantly lower levels of the IFNβ compared to WT in media (Fig. 6a, One-tailed Student’s *t*-test, *n* = 3, *p* < 0.05). Additionally, the mRNA levels of interferon regulatory factor 7 (*IRF7*) and interferon stimulate gene 56 (*ISG56*) were both lower in MEFs infected with 2B mutants than WT (Fig. 6b). If these 2B mutants have a higher capacity to suppress innate immune responses in vivo, it would be expected that these mutants could replicate more effectively in infected animals. Indeed, in innate immune-competent Tg21 mice, 2B mutants (I2V, I6V, and I6M) exhibit increased replication levels in muscle, kidney, and brain compared to WT (Fig. 6c) and increased virulence in infected animals (Fig. 6d).

Taken together, these results suggest that the high fitness alleles that accumulated in 2B during adaptation to IFN pretreatment in cell culture increase the rate of replication, inhibit protein secretion and counteract IFN responses. This suggests that 2B regulates virulence in vivo by modulating IFN responses (Figs. 5 and 6). Although the mutations identified in cell culture differ from those in the organ-resident virus populations observed in vivo, it is consistent with the concept that the ability to rapidly adapt is central to the fitness of viral populations. Facing variable selective environments, the adaptive capacity of viral populations enables rapidly access to evolutionary solutions.

## Discussion

The selective environment encountered by the viral populations in vivo is dynamic and heterogeneous. Here we report that, during WT poliovirus infection, this heterogeneous selective environment drives the development of tissue-specific patterns of diversity in the organ-resident virus populations. Importantly, none of the adaptive alleles are fixed in the population. As a result, the virus maintains the master consensus sequence. In contrast to classical evolutionary theory, where beneficial alleles compete to fix in the population, we propose that the population master sequence has been positioned in sequence space to optimize its access to distinct genetic neighborhoods. These functional subpopulations then cooperate to overcome tissue-type and cell-type specific selective pressures (Fig. 4d)^36^. In the contrary, GD virus, which is engineered with limited evolutionary capacity, is unable to establish similar population compositions to WT. This is correlated with the IFN-dependent attenuation of GD in vivo, suggesting that the rapid adaptive capacity of viral populations is required to overcome early innate immune responses. Of note, while the observations presented here suggest a central role of diversity in counteracting innate immunity, other challenges, such as those imposed by specific cell-type and physicochemical environments, are expected to be present during infection.

Our experiments suggest that the diversity and genetic composition of poliovirus population, and their spatial and temporal dynamics, are virulence determinants. Viral populations rapidly and reproducibly adapt to distinct challenges present in the host environment, thereby locally regulating specific viral functions in response to selections. Our findings provide mechanistic insight into the role of the RNA virus population diversity, subpopulation cooperation, and group selection during in vivo infection. The maintenance of genetic diversity and structure in a viral population facilitates successful adaptation by providing potential solutions to the selective challenges encountered in distinct environments, tissues or organisms. Adaptive alleles can only rapidly accumulate with effective recombination to alleviate the deleterious mutational load. We show, through the modulation of these parameters, that the balance of mutation and recombination, is finely tuned and determines the outcome of infection. These forces are also expected to play an important role across larger scales as well, such as in host-to-host transmission, a process central to virus survival.

Acute infection is characterized by rapid evolution to specific pressures, and our results suggest that tampering with the adaptive capacity of a virus may provide a means to alter the outcome of infection. This presents a number of possibilities for therapeutic strategies and rational vaccine design. A better understanding of the correlation between population allele composition and tissue adaptation may allow the design of viruses with specific host and tissue tropism. Moreover, linking the population dynamic effects of mutation and recombination rates to specific viral polymerases may be fundamental for our understanding of viral infection and dynamics in nature, and may help to explain why some viruses are more virulent than others, or how attenuated strains (like the Sabin PV vaccine strain) evolve to more pathogenic phenotypes.

## Methods

### Cells, plasmids and viruses

HeLaS3 cells (ATCC, CCL-2.2), L929 (ATCC, CCL-1), MG63 (ATCC, CRL-1427), COS7 (ATCC, CRL-1651), L20B cells were obtained from National Institute for Biological Standards and Control (NIBSC, United Kingdom) and primary embryo fibroblast cells (MEFs) isolated from 12.5 to13.5 days pregnant Tg21PVR mice. L20B and MEFs were cultured in DMEM high glucose/F12 medium (UCSF cell culture facility) supplemented with 10% newborn calf serum (Sigma) and 1xpenicillin/streptomycin/glutamine (100×PSG, Gibco). The Mahoney strain of poliovirus Type 1 was used as WT virus in this study. Viral RNA-dependent RNA polymerase (RdRp) mutant viruses (G64S/D79H (GD)), and 2B mutants (I2V (1032 Ile->Val, nucleotide position, 3836–3838 ATC->GTG), I6V (1036 Ile->Val, nucleotide position, 3848 A->G), I6M (1036 Ile->Met, nucleotide position, 3850 A->G)) were modifications from WT Mahoney type 1 poliovirus (PV) strain. To generate these mutant viruses, T7 polymerase was used to generate in vitro transcribed (IVT) viral RNA derived from corresponding linearized prib(+)XpA Mahoney plasmid. The resulting 10 μg IVT RNAs were electroporated into 8 × 10^6^ HeLaS3 cells. Viruses were harvested at 24 h to generate P0 virus stocks. P0 stocks were amplified once in HeLaS3 cultured in 2% serum media approximately m.o.i=10 to generate a passage 1 (P1) stocks. In all subsequent experiments P1 stocks were used.

### Identification of recombination deficient poliovirus

To screen for recombination-deficient poliovirus, a GFP gene was cloned into the poliovirus Mahoney genome (GenBank: NC_002058.3) between the structural proteins and non-structural proteins flanked by 2 A cleavage sites (2A-GFP-2A). The resulting polio-GFP (PV-GFP) virus was passaged using limited dilution at each passage in HeLaS3 cells in 96 wells. The GFP retention ratio was measured in plate reader. A viral clone with reduced recombination was selected based on a high GFP retention ratio. Sanger sequencing was performed to sequence the viral clone, a single amino acid mutation, an aspartic acid 79 to histidine (D79H) in RNA-dependent RNA polymerase (RdRp) was identified. Then, D79H mutation was cloned back to the poliovirus genome and the GFP retention experiment was repeated. The result confirmed that D79H confers a viral phenotype deficient in recombination. The GFP retention rate for D79H was approximately 60% compared to 10% for WT^26^.

### CRE-REP recombination assay

Two non-viable, (PV^cre^) and IVT viral RNAs were used in a previously described recombination assay^29^ (Fig. 1a). However, viable progeny can be generated upon recombination between the non-viable viral RNAs. The first variant of IVT RNA (PV^cre^) contained a mutation in the cis-acting replication element (CRE) within the 2 C coding region. This mutation impairs RNA synthesis and, therefore, does not produce viable progeny. A second viral RNA lacking capsid proteins (Rep1L) is replication competent but is unable to produce viable progeny due to the lack of structural proteins. For homologous recombination, we used type 1 poliovirus Mahoney strain as the genetic backbone. The synthesis-restricting mutation in PV^cre^ is A4472G (referred below as PV1^cre^)^37^. For non-homologous recombination, we used Sabin 3 strain of poliovirus, with eight synthesis-restricting synonymous substitutions within CRE (referred below as S3B^cre^)^38^.

The second variant of IVT RNA is a sub-genomic replicon (Rep1L) where capsid is replaced with luciferase. Rep1L also does not produce viable progeny on its own, because it cannot encode capsid. Following co-transfection PV1^cre^ and Rep1L (or PV1^cre^ and Rep1L containing D79H mutant) into permissive L929 cells, viable progeny is produced only if recombination of the two defective variant RNAs takes place between the structural proteins (capsid) and CRE. The same assay was used for non-homologous recombination, where PV1^cre^ was replaced by SB3^cre^. Thus, either 5 μg of each variant RNAs alone or 10 μg of two variants together were transfected by electroporation into approximately 4 × 10^6^ of permissive L929 cells. Then, cells were incubated at 37 °C for 24 h. The standard TCID_50_ assay was performed to titer the virus stocks.

### Virus growth curve and qRT-PCR analysis of viral RNA replication kinetics

2.5 × 10^5^ L20B cells were seeded in 12-well plates. On the following day, cells were washed twice with PBS and were infected with virus in 200 μl serum-free media at m.o.i=0.1 (*n* = 3, three replicate wells were used for each virus at each time point). Following a 40 min incubation at 37 °C, each well was washed twice with PBS, and cells were covered with fresh complete media. At each indicated time point, the corresponding plate was removed and frozen at −80 °C. Following three freeze-thaw cycles of the plates, standard plaque assays were performed on monolayer HeLaS3 cells grown in a 6-wells plate (10^6^ HeLaS3 cells per well). For qRT-PCR assay, RNA was extracted from 200 μl cell-free viral supernatant for each virus at each time point (0–12 h) by purelink RNA mini kit (Life Technologies), poliovirus specific primer (primer-RT) was used to prime cDNA synthesis on 1/4 of each RNA preparation (4.5 μl) in a 10 µl volume with reverse transcriptase III (Invitrogen). cDNA were diluted and qPCR primers as described^39^, and 2 μl of this diluted cDNA was analyzed by RT-PCR by using 2×Fast Mastermix (KAPA Biosystems). The cycling condition used were 95 °C for 5 min, 40 cycles of 95 °C for 5 s, and then 60 °C for 30 s^40^.

### Growth competition assays

Monolayer L20B cells (10^6^ cells per well) were grown in 6-well plates and were infected by equal amounts of two virus strains with the total m.o.i = 0.01 at a ratio 50%:50%. Cells were infected for 8 h (Passage 1). The virus stocks were frozen at −80 °C. Following 3 cycles of freezing and thawing, 1/100th of the volume (20 μl) of passage 1 stocks (2 ml) were transferred to fresh cells at the next passage (Passage 2). This process was repeated for the total of four passages. Total RNA was extracted from virus stocks using Trizol (Ambion). Poliovirus genome-specific primer was used to prime cDNA synthesis with 1/10th volume (2 μl) of the total RNA by in vitro transcriptase III (SS III, Invitrogen). RT-PCR was performed by using specific poliovirus primers. Reactions were subjected to 40 cycles of 95 °C for 30 s, 58°C for 30 s, 72 °C for 30 s, sanger sequencing the PCR products of passage 1 (P1) and passage 4 (P4).

### Interferon β (IFNβ) production measured by ELISA and qRT-PCR

2.5 × 10^5^ cells/well MG63 cells were seeded into 12 well plates, overnight. Cells were infected by WT poliovirus, 2BI2V, 2BI6V or 2BI6M for 40 min at m.o.i=20, then at 5.5 and 7.5 h postinfection, the supernatant was collected to measure interferon production by ELISA (VeriKine-HS Human Interferon Beta Serum ELISA Kit, PBL Assay Science). For experiments performed on murine embryos fibroblast cells (MEFs), cells were isolated from 12.5–13.5 day old embryos. MEF monolayers were seeded at 2.5 × 10^5^ cells per well in 12-well plates, infected by WT poliovirus, 2BI2V, 2BI6V, or 2BI6M for 40 min at m.o.i=0.1. At 72 h postinfection, supernatant was collected to measure IFNβ induction by ELISA (VeriKine-HS Murine Interferon Beta Serum ELISA Kit, PBL Assay Science). Then medium was aspirated and washed twice with PBS, total RNA was extracted with 200 μl Trizol Reagent (Ambion) and treated with Dnase1. 1 µg Dnase1-treated RNA was used to synthesize cDNA by iScript cDNA Synthesis kit (Bio-rad). cDNA was quantified by real-time qRT-PCR by 2×Fast Mastermix (Kapa Biosystems). Sequencing primers showed as Supplementary Table 2. The thermocycling conditions for *IL6, IL8, TNFα, RIG-I*, and *β-actin* was 95 °C for 5 min, 45 cycles of 95 °C for 5 s, and then 55 °C for 30 s and 72 °C for 20 s; condition for *IRF7* and *ISG56* was 95 °C for 5 min, 45 cycles of 95 °C for 5 s, and then 58 °C for 30 s and 72 °C for 20 s. The *β-actin* gene was used for normalization of murine experiments and *GAPDH* was used for human cell experiments. Data were analyzed using the comparative ΔΔCt method using the following formula: ΔCt=Ct (target)−Ct (normalizer). The comparative ΔΔCt calculation involved finding the difference between the target (GD, 2B mutant or WT) ΔCt and the baseline (PBS) ΔCt. Fold changes in the expression of specific mRNA compared with *β-actin or GAPDH* was calculated as 2^-(ΔΔCt)^. Student *t*-test, n.s. indicates *P* > 0.05. *P ≤ 0.05, **P ≤ 0.01, ***P ≤ 0.001.

### Interferon α response sensitivity assay

Approximately 2.5 × 10^5^ monolayer HeLaS3 cells were pre-treated with 500 IU/ml of IFN α(Universal type I IFN alpha, PBL Assay Science) for 24 h, washed twice with PBS, then infected by virus at m.o.i=0.01 for 40 min. Virus was removed and cells were washed with PBS and supplemented with fresh medium and incubated at 37 °C for 24 h. Plates were then frozen at −80 °C. Following 3 freeze and thaw cycles, plaque assays were performed on HelaS3 cells to quantify virus production.

### Inhibition of protein secretion activity

We transfected 50 μg reporter plasmid carrying EMCV-IRES-driven SEAP reporter into ~5 × 10^6^ monolayer COS7 cells by Lipofectamine 2000^34^. After 6 h transfection, cells were trypsinized and seed into 12 wells plate (2.5 × 10^5^ cells per well). On the following day, cells were infected with poliovirus: WT, 2B I2V, 2B I6V, 2B I6M at m.o.i=10. Media were collected as indicated time-points. SEAP activity were measured by Great EscAPe SEAP Chemiluminescence Kit 2.0 Kits (Clontech).

### Infection of susceptible mice

We followed protocols approved by the UCSF Institutional Animal Care and Use Committee for the mouse studies. In these experiments, 5 to 6-weeks-old Tg21PVR (Tg21) or Tg21 PVR α/β IFN receptor knockout (IFNAR^−/−^) both male and female mice were used and infected under anesthesia. Tg21 and IFNAR^−/−^ were kindly provided by Professor Julie Pfeiffer of the University of Texas Southwestern Medical Center, and originally were generated by Dr. Satoshi Koike of Tokyo Metropolitan Institute for Neuroscience. For mice survival studies, mice were injected by intra-peritoneal injection (I.P., 100 μl per mouse) or by intra-muscular (I.M., 50 μl of inoculum administered in each hind leg) with serial dilutions of each virus, respectively (10 mice per group). Mice were monitored daily for the onset of paralysis and were euthanized when death was imminent. For tissue distribution studies, we injected Tg21 mice (5 mice per group) with viral supernatant 10^6^ PFU per mouse by I.P. route, 10^4^ PFU per mouse by I.M. and intra-cranial (I.C.) route, respectively (4 mice per group). IFNAR^−/−^ mice were inoculated by intra-peritoneal route with 10^4^ PFU per mouse (5 mice per group). Whole organs were collected from infected mice and homogenized in 1 ml serum-free media. Viral supernatants were collected from the tissue homogenates, following three freeze-thaw cycles, and centrifuged at 5000 g for 10 min in a bench top centrifuge at 4 °C. Regular plaque assays were performed on HeLaS3 cells to titer viral supernatants from tissues.

### In vivo RNA deep sequencing (RNA-Seq) of host transcriptomes and viral populations

Five to 6-weeks-old Tg21 mice (three mice per group, per time point) were infected with mock (PBS) or different doses of WT virus: WTC (10^6^ PFU WT virus per mouse), WTB (10^7^ PFU WT virus per mouse) or 10^8^ PFU GD. Organs were collected at one and 3 days postinfection. ~ 100 mg of each organ from infected Tg21 mice were homogenized in 1 ml Trizol reagent. Total RNA was extracted, and then treated with DNase1. 10 µg DNase1 treated RNA was purified by poly-A beads (Bioo scientific). For viral population sequencing, tissues were homogenized in 1 ml serum-free medium. RNAs were extracted from supernatants by Zymo-viral RNA extract kit (Zymo Research). The viral RNAs were purified by poly-A beads (Bioo scientific). RNA-Seq libraries were made using KAPA stranded RNA-Seq kit (Kapa Biosystems). Viral and host RNA-Seq libraries were sequenced by Illumina Hiseq 4000.

Pairwise genetic distances between sequenced viral populations were computed as the Weir-Reynolds distance for all alleles detected by RNA-Seq^41^. Multidimensional scaling and visualization of viral sequence and gene expression trajectories was performed in R with the “cmdscale” function and the “ggplot2” package. Differential gene and transcript expression analysis of RNA-Seq experiments with TopHat and Cufflinks^42^. Host gene expression profiles for (*N* = 565) were clustered across all times and conditions using the “ward.D2” algorithm based on pairwise Euclidian distances between expression profiles. After dendrogram visualization, 5 clusters were assigned using “cutree” in R. Sequence diversity was computed as Shannon’s entropy^43^ using a log base 4, to scale entropy values between 0 and 1.

### CirSeq of poliovirus populations

Each viral population was generated by infecting 10^7^ monolayer HeLaS3 cells at m.o.i=0.1 for single round replication cycle 8 h. For IFN treated condition, we pre-treated 10^7^ monolayer HeLaS3 cells with 10 ml, 1 IU/ml IFNβ (PBL Assay Science) for 14 h, PBS wash twice before virus infection. We performed seven passages for each virus strain. To amplify each viral population, 10^7^ monolayer HeLaS3 cells were infected at m.o.i=10. Virus was harvested after completely cytopathic effect (at postinfection 8 to 9 h). CirSeq libraries were produced by following the protocol described previously and sequenced by Illumina HiSeq 2500^27, 28^.

### Data analysis and statistical analysis

Data were presented as mean ± s.d. Statistical analysis was performed using GraphPad Prism (GraphPad Software). Statistical significance was calculated using a Student’s *t*-test and *p *< 0.05 was considered significant. 50% lethal dose (LD50) was calculated as described by the Reed and Muench method. Significance is noted with asterisks as described in the figure legends. Animal experiments were not blinded or randomized, and no animals or samples were removed as outliers from the analysis.

### Fitness calculation of CirSeq data

All CirSeq viral populations sequencing data was analyzed by the CirSeq V2 pipeline. Sequence diversity was computed as Shannon’s entropy^43^ using a log base 4, to scale entropy values between 0 and 1. For fitness calculations, the deviation from normality in standard deviation (s.d.) units was computed by Binomial Bayesian regression as described^28^. The comparison of IFN treated and untreated poliovirus populations was performed for approximated 5 passages as follows: we restored frequencies of the passages 4 and 7 as the averages of the neighboring passages 3, 5 for passage 4 and 6, 8 for passage 7, and then coverage/mutation counts were estimated from frequencies and average coverages of two neighboring passages for passages 4 and 7. We calculated the normalized fitness for PV and PV + IFN datasets.

### Code availability

Code supporting this analysis has been deposited in the GitHub repository (https://github.com/ptdolan/Xiaoetal_2017_analysis).

### Data availability

Raw deep-sequencing read data have been deposited in the NCBI BioProject database with accession code PRJNA383905. The authors declare that all other data supporting the findings of this study are available in the article and its Supplementary Information files, or from the Stanford Digital Repository (https://purl.stanford.edu/pc148ks8466).

## Electronic supplementary material

Supplementary Information
