# Supplementary material for: Poliovirus intrahost evolution is required to overcome tissue-specific innate immune responses
Source: Nat Commun. 2017 Aug 29;8:375. doi: 10.1038/s41467-017-00354-5 (PMC5575128; doi:10.1038/s41467-017-00354-5)
Supplement: Supplementary file 1 — Supplementary Information [file 41467_2017_354_MOESM1_ESM.pdf]

File name: Supplementary Information
Description: Supplementary Figures, Supplementary Tables and Supplementary References

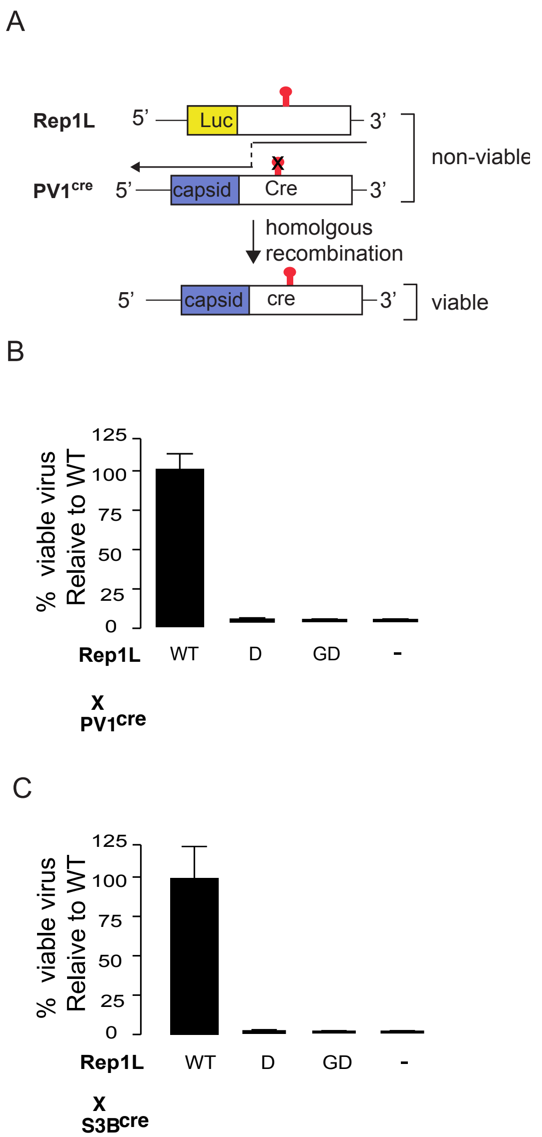

Supplementary Fig.1. A single amino acid substitution D79H within the poliovirus RNA dependent RNA polymerase (RdRp) reduces the recombination rate.

(**A**) Schematic representation of the CRE-REP recombination assay (*1*). *In vitro* transcript (IVT) RNAs were made from corresponding linearized plasmids PV1^{cre}, Rep1L and SB3^{cre}. PV1^{cre} contains mutant and inactivating the *cis*-acting replication element (CRE), which prevents positive strand viral RNA synthesis (*2-3*). Sub-genomic replicon (Rep1L) does not encode structural proteins. Thus, neither construct alone can produce viable progeny. Following co-transfection of PV1^{cre} and Rep1L into permissive cells (L929

cells), viable progeny is produced only if recombination of the two defective PV1^{cre} and Rep1L RNAs takes place at any site between the structural proteins (capsid) and CRE.

(**B**) Co-transfection of PV1^{cre} and Rep1L or Rep1L with D79H mutation in RdRp into permissive L929 cells. The titer of viable progeny (TCID50 per ml) was measured by the standard TCID50 assay in HelaS3 cells and normalized to Rep1L carrying a wild-type RdRp (WT).

(**C**) Using a similar CRE-REP assay, we also examined recombination between poliovirus Sabin3 strain (SB3^{cre}) and type 1 Rep1L. In this experiment, Sabin3 RNA (SB3^{cre}) contains a mutation within the *cis*-acting replication element (CRE) and Rep1L [or Rep1L carried a mutation in the recombination determinant D79H (D) or G64S/D79H (GD)]. For details see Methods.

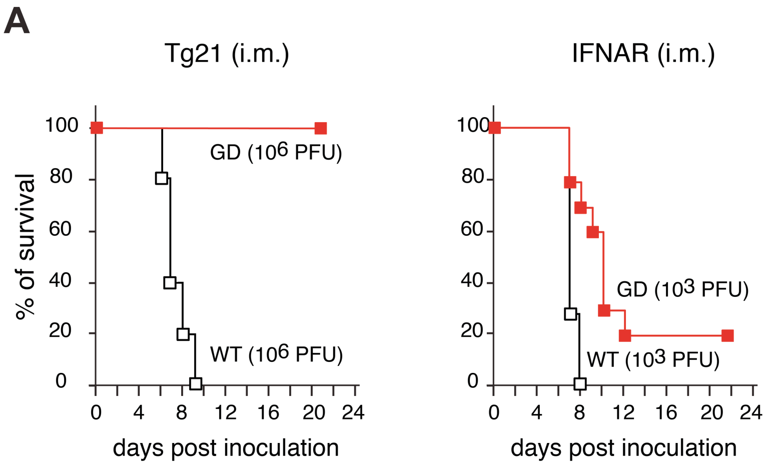

Supplementary Fig. 2. Intra-muscular inoculation of wild type and GD poliovirus.

Kaplan-Meier survival curves in tg21 PVR (Tg21) or Tg21 PVR interferonα/β receptor knockout mice (IFNAR) following intra-muscular (i.m.) inoculation. 10^6 PFU virus per tg21 mouse or 10^3 PFU virus per IFNAR were injected into mice (n=10, the number of mice is ten per group, Log-rank (Mantel-Cox) test). Wild type virus population (WT, open squares), GD virus population (squares with red).

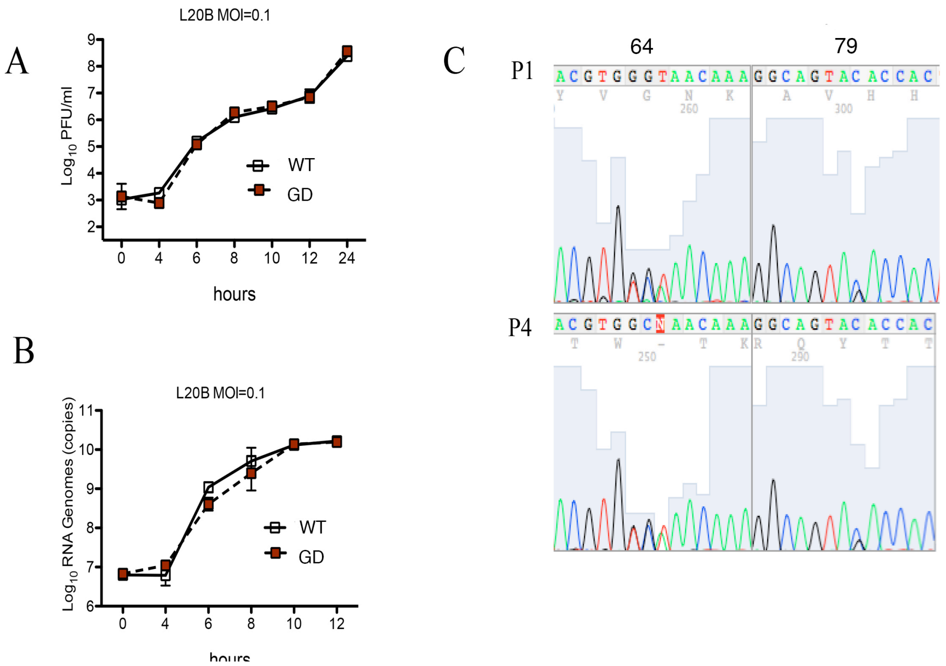

Supplementary Fig. 3. Replication and fitness effect of viral strains in murine L20B cell line.

(**A**) Viral growth kinetics in murine cell line L20B. L20B cells were infected by GD or wild type virus at m.o.i=0.1. Titers were measured by plaque assays in HelaS3 cells.

(**B**) Viral RNA synthesis. Viral RNA replication in L20B cells at m.o.i=0.1. Measured by RT-qPCR. Data presented as logarithm, mean ± s.d. RNA genome copies per ml. No significant difference was observed at any point of the time course. Student t test, n=3, three replicates for each time, for each viral strain.

(**C**) Competition assay in L20B cells. Cells were infected with total m.o.i= 0.01, at a ratio of GD with wild type virus of ~ 50%: 50%. The virus mixture was passed under the same conditions four times (method). Ratios of GD and WT viruses were estimated by sequencing over the region containing the G64S (64) and D79H (79) mutations. No significant difference was observed on the sequence chromatograms of passage 4(P4) compare to passage 1(P1).

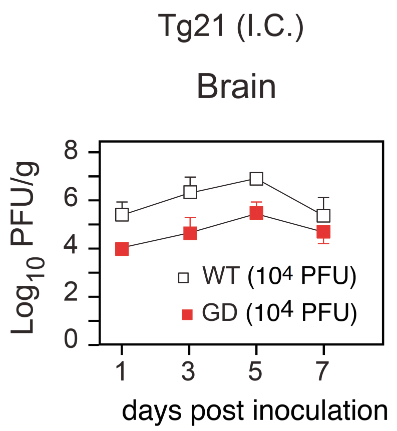

Supplementary Fig. 4. GD population replicates in brain in tg21 mice following direct inoculation in that tissue. Tg21 mice were infected by intra-cranial (I.C.) inoculation route with 10^4 PFU per mouse . Brain was collected at indicated time points and virus titers measured by plaque assay on HelaS3 cells. Data are shown as logarithm, mean ± s.d. (n=4, the number of mice is four per group).

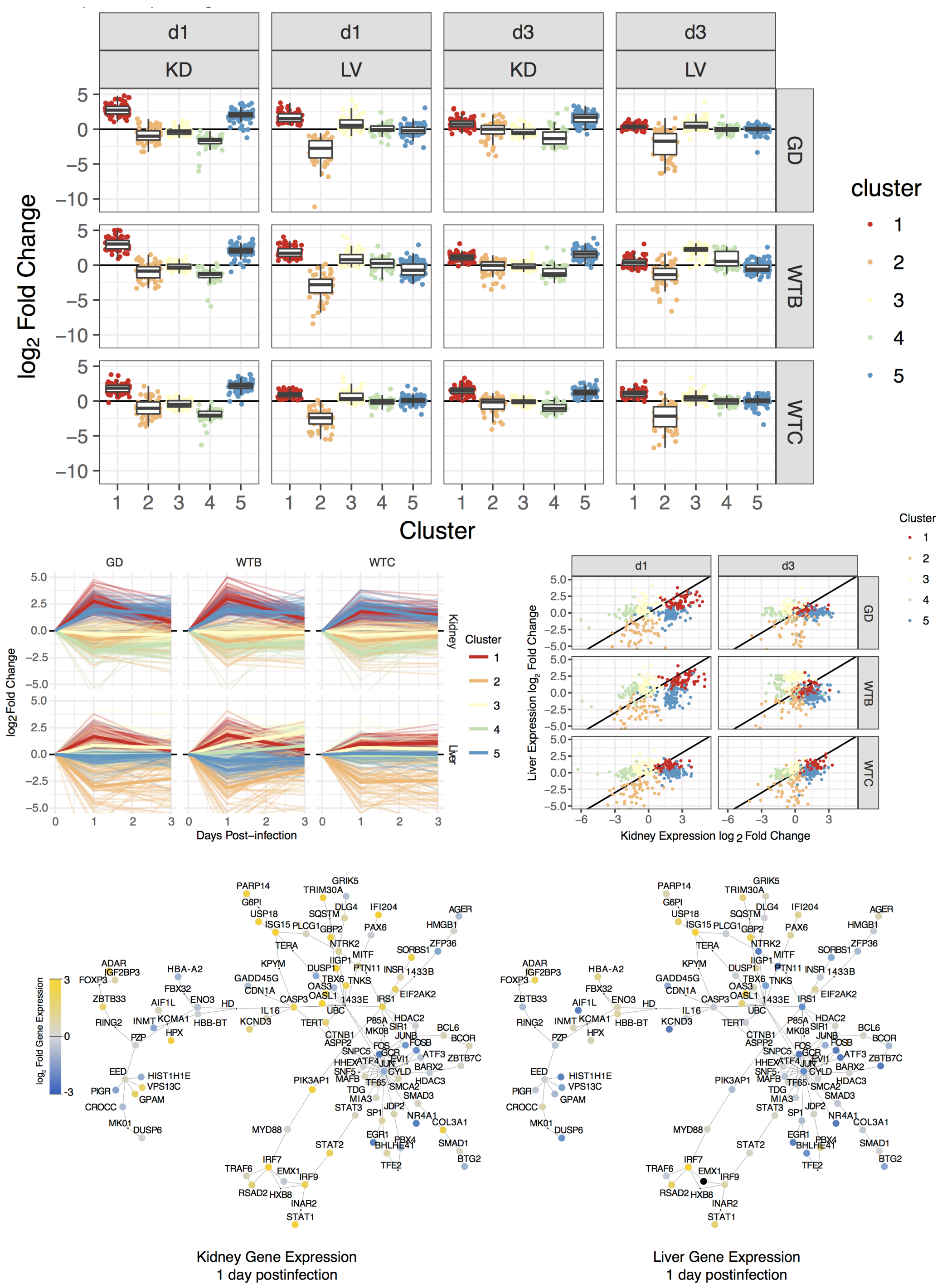

d1
d3
KD
LV
GD
WTB
WTC
log2 Fold Change
Cluster
cluster
1
2
3
4
5
Days Post−infection
Kidney
Liver
Liver Expression log2 Fold Change
Kidney Expression log 2 Fold Change
log2 Fold Gene Expression
Kidney Gene Expression
1 day postinfection
Liver Gene Expression
1 day postinfection

Supplementary Fig.5. Poliovirus-specific interferon regulatory factors (IRFs) and interferon-stimulated genes (ISGs) induction in Tg21 mice. Tg21 mice were infected with 10^6 PFU (WTC) or 10^7 PFU (WTB) of wild type poliovirus (PV type 1, Mahoney strain) or 10^8 PFU of GD mutant poliovirus administered by I.P. route and compared to mock-infected mice (PBS) (n=3, the number of mice is three per group per time-point). Mouse organs were collected at one and three days post-infection. (A) Organ-specific gene expression and significance levels (q-value) across all experimental conditions were clustered by hierarchical clustering using the 'Ward.D2' algorithm based on Euclidian distance. After dendrogram visualization, clusters were assigned using cutree to yield 5 clusters (Methods). (B) Individual gene trajectories are shown for all infection conditions. Notably, the gene expression responses to infection in WTB (10^7 PFU) and GD are similar. (C) Scatter plots comparing gene cluster expression in liver and kidney at 1 and 3 days postinfection. (D) Largest component of functional interaction network built from genes with altered expression during infection. The network is highly enriched in antiviral and innate immune genes, which are much more highly upregulated in kidney (left) than in liver (right) at day 1 postinfection.

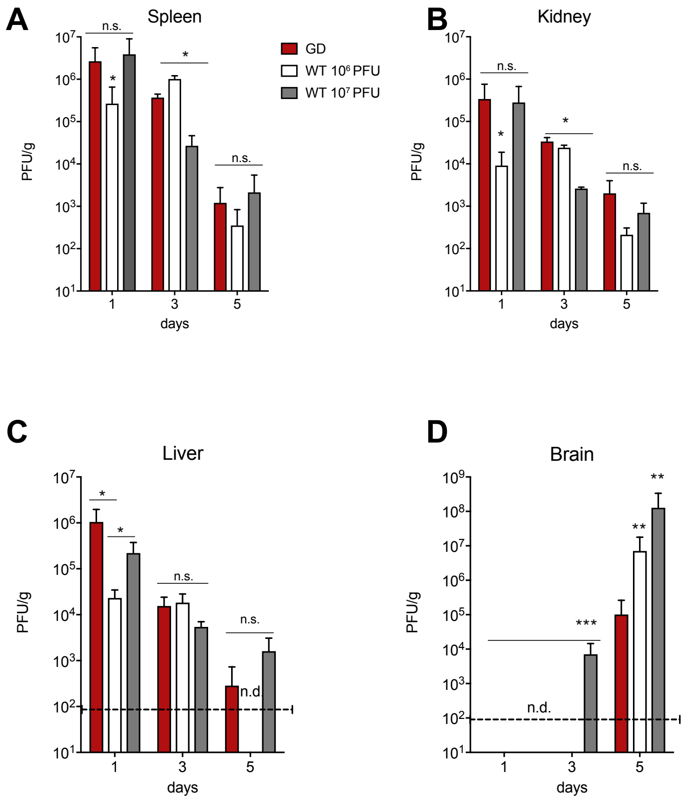

Supplementary Fig.6. Virus titer from tg21 mice infected with different infection doses of wild type and GD per mouse by I.P. route. WT 10^7 PFU: 10^7 PFU WT virus per mouse, WT 10^6 PFU : 10^6 PFU WT virus per mouse. GD: 10^8 PFU virus per mouse. Regular plaque assays were performed in HeLaS3 cells. Data are shown as logarithm, mean ± s.d., PFU per gram of tissue. Student's t-test. Limited detection level is 100PFU per gram tissue. The number of mice for each group and each time-point is three (n=3). n.s. indicates $P > 0.05$, $*P \leq 0.05$, $**P \leq 0.01$, $***P \leq 0.001$.

Supplementary Table.1 Beneficial mutations were identified in Hela cells.

Table 1: Beneficial mutations (>25 s.d. above neutrality, N=27) identified in passage experiments with WT, GD, and WT treated with interferon beta. Mutations in the 2B protein are shown in **bold.**

Position (NT)	Position (ORF)	WT NT	Mutant NT	Viral Protein	Subtype	WT Codon	Mut Codon	WT Residue	Mut Residue	SDs above neutrality	Sample
120	–	C	T	5'UTR	–	–	–	–	–	59.6853	WT PV−A
2728	662	G	A	VP1	S	CGG	CGA	R	R	66.5817	WT PV−B
3265	841	A	T	VP1	NS	AAA	AAT	K	N	34.7531	WT PV−B
3719	993	A	T	2A	NS	ATA	TTA	I	L	198.5020	WT PV−B
3810	1023	A	G	2A	NS	TAC	TGC	Y	C	34.8669	WT PV−A
**3836**	**1032**	**A**	**G**	**2B**	**NS**	**ATC**	**GTC**	**I**	**V**	**30.3994**	**WT PV−B**
**3848**	**1036**	**A**	**G**	**2B**	**NS**	**ATA**	**GTA**	**I**	**V**	**154.3500**	**WT PV−B**
**3848**	**1036**	**A**	**G**	**2B**	**NS**	**ATA**	**GTA**	**I**	**V**	**43.6812**	**WT PV−A**
**3848**	**1036**	**A**	**G**	**2B**	**NS**	**ATA**	**GTA**	**I**	**V**	**109.6810**	**WT PV+IFN**
**3850**	**1036**	**A**	**G**	**2B**	**NS**	**ATA**	**ATG**	**I**	**M**	**28.9399**	**WT PV−B**
**3850**	**1036**	**A**	**G**	**2B**	**NS**	**ATA**	**ATG**	**I**	**M**	**62.7006**	**WT PV−A**
**3884**	**1048**	**A**	**G**	**2B**	**NS**	**ACT**	**GCT**	**T**	**A**	**79.5745**	**WT PV−B**
**3884**	**1048**	**A**	**G**	**2B**	**NS**	**ACT**	**GCT**	**T**	**A**	**41.0824**	**WT PV−A**
**3893**	**1051**	**A**	**C**	**2B**	**NS**	**ATT**	**CTT**	**I**	**L**	**68.4935**	**WT PV−B**
**3893**	**1051**	**A**	**C**	**2B**	**NS**	**ATT**	**CTT**	**I**	**L**	**87.2271**	**WT PV−A**
**3942**	**1067**	**C**	**T**	**2B**	**NS**	**ACT**	**ATT**	**T**	**I**	**75.8518**	**WT PV−B**
**3942**	**1067**	**C**	**T**	**2B**	**NS**	**ACT**	**ATT**	**T**	**I**	**93.3319**	**WT PV+IFN**
**4017**	**1092**	**C**	**T**	**2B**	**NS**	**ACA**	**ATA**	**T**	**I**	**59.4925**	**WT PV−B**
4187	1149	T	G	2C	NS	TCA	GCA	S	A	33.8123	WT PV−B
4199	1153	T	A	2C	NS	TCA	ACA	S	T	58.4998	WT PV−A
4354	1204	C	A	2C	NS	TTC	TTA	F	L	28.9896	WT PV−B
5026	1428	C	T	2C	S	TAC	TAT	Y	Y	51.7420	G64SD79H
5431	1563	G	A	VPg	S	AAG	AAA	K	K	26.7296	WT PV−A
6281	1847	G	A	3D	NS	GAT	AAT	D	N	30.5566	WT PV−B
6587	1949	A	G	3D	NS	AAC	GAC	N	D	33.7708	WT PV−B
6778	2012	C	T	3D	S	TAC	TAT	Y	Y	35.3816	G64SD79H
6781	2013	C	T	3D	S	ATC	ATT	I	I	194.8880	WT PV−B

Supplementary Table.2 The sequences of qRT-PCR Primers

	Name	Sequence
Murine	IRF7_Forward	atc aga agc agc tgc act aca c
Murine	IRF7_Reverse	tac acc ttg cac ttg ccc ata
Murine	ISG56_Forward	ggc aggacaatgtgcaagaat
Murine	ISG56_Reverse	agcagtcagtagtttcctcc
Murine	β-actin_ Forward	ccg taa aga cct cta tgc caa
Murine	β-actin_Reverse	agg agc cag agc agt aat ct
Human	IL6_Forward	AAG AGT AAC ATG TGT GAA AGC
Human	IL6_Reverse	CTA CTC TCA AAT CTG TTC TGG
Human	TNFα_Forward	CAGAGGGAAGAGTCCCCCAGG GACC
Human	TNFα_Reverse	CCTTGGTCTGGTAGGAGACGGCGATG
Human	IL8_Forward	ATGACT TCCAAGCTGGCCGTGGCT
Human	IL8_Reverse	TCTCAGCCCTCTTCAAAAACTTCTC
Human	GAPDH_Forward	CTGGGCTACACTGAGCACCAG
Human	GAPDH_Reverse	CCAGCGTC AAAGGTGGAG
Human	RIG-I_ Forward	AGTGAGCATGCACGAATGAA
Human	RIG-I_Reverse	GGGATCCCTGGAAACACTTT

Supplementary References:

1. K. Lowry, A. Woodman, J. Cook, D. J. Evans, Recombination in Enteroviruses Is a Biphasic Replicative Process Involving the Generation of Greater-than Genome Length 'Imprecise' Intermediates. *Plos Pathogens* **10**, (Jun, 2014).
2. E. Rieder, A. V. Paul, D. W. Kim, J. H. van Boom, E. Wimmer, Genetic and biochemical studies of poliovirus cis-acting replication element cre in relation to VPg uridylylation. *Journal of virology* **74**, 10371 (Nov, 2000).
3. I. G. Goodfellow, D. Kerrigan, D. J. Evans, Structure and function analysis of the poliovirus cis-acting replication element (CRE). *Rna* **9**, 124 (Jan, 2003).
